# Supplementary figures and images for: Identification of key genes and biological processes contributing to colitis associated dysplasia in ulcerative colitis
Source: PeerJ. 2021 Apr 27;9:e11321. doi: 10.7717/peerj.11321 (PMC8086577; doi:10.7717/peerj.11321)

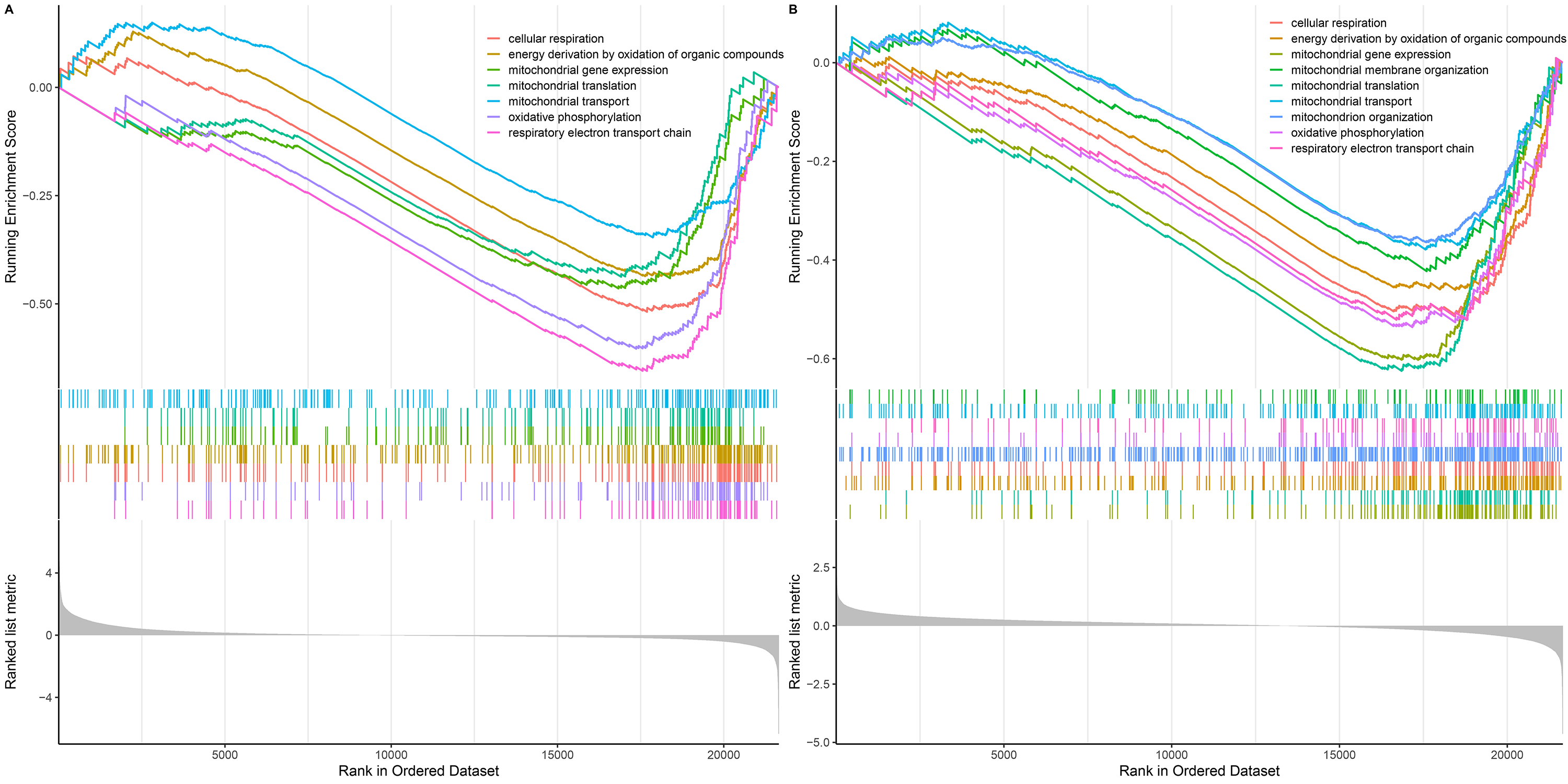

Supplement: Supplemental Information 1 — (A) GSEA analysis of mitochondrial function-related GO-BPs between UC and control groups. (B) GSEA analysis of mitochondrial function-related GO-BPs between CAD and control groups. [file peerj-09-11321-s001.png]
