# Supplementary material for: Identification of key genes and biological processes contributing to colitis associated dysplasia in ulcerative colitis
Source: PeerJ. 2021 Apr 27;9:e11321. doi: 10.7717/peerj.11321 (PMC8086577; doi:10.7717/peerj.11321)
Supplement: Supplemental Information 2 [file peerj-09-11321-s002.docx]

|  | Controls (n=15) | Ulcerative colitis (n=20) | Colitis associated dysplasia  (n=6) | Chi-squared test p-value |
| --- | --- | --- | --- | --- |
| Gender |  |  |  |  |
| Male | 4 | 7 | 4 | 0.22 |
| Female | 11 | 13 | 2 |  |
| Age (median, IQR) | 52 (34) | 42 (30) | 49 (28) |  |
| Disease duration |  |  |  |  |
| <10 years | - | 13 | 2 | 0.17 |
| >10 years | - | 7 | 4 |  |
| Mayo score (median, IQR) | - | 6 (4) | 1.5 (5) |  |
| Mayo endoscopic score (median, IQR) | - | 2 (0) | 0.5 (2) |  |
| Smoking status |  |  |  |  |
| Smoking | 2 | 3 | 0 | 0.61 |
| Nonsmoking | 13 | 17 | 6 |  |
| Daily medication |  |  |  |  |
| Systemic 5-ASA | - | 17 | 2 | 0.36 |
| Topical 5-ASA | - | 3 | 0 |  |
| Systemic steroids | - | 3 | 0 |  |
| Topical Steroids | - | 2 | 0 |  |
| Thiopurines | - | 5 | 2 |  |
| Infliximab | - | 1 | 0 |  |
| none | - | 2 | 2 |  |

**Table S1.** **Clinical details of GSE47908**

, p-value cannot be calculated because of limited information. IQR, interquartile range.
