# Supplementary material for: Identification of key genes and biological processes contributing to colitis associated dysplasia in ulcerative colitis
Source: PeerJ. 2021 Apr 27;9:e11321. doi: 10.7717/peerj.11321 (PMC8086577; doi:10.7717/peerj.11321)
Supplement: Supplemental Information 3 [file peerj-09-11321-s003.docx]

**Table S2. Top 6 GO-BP terms of each module**

| **Module**  **(Total gene number)** | **ID** | **GO-BP terms** | **Gene Numbers** | **Rich Factor** |
| --- | --- | --- | --- | --- |
| Turquoise  (1867) | GO:0097501 | stress response to metal ion | 9 | 0.53 |
|  | GO:0071294 | cellular response to zinc ion | 10 | 0.43 |
|  | GO:0010043 | response to zinc ion | 15 | 0.28 |
|  | GO:0010273 | detoxification of copper ion | 8 | 0.53 |
|  | GO:1990169 | stress response to copper ion | 8 | 0.53 |
|  | GO:0043161 | proteasome-mediated ubiquitin-dependent protein catabolic process | 55 | 0.13 |
| Blue  (1252) | GO:0045216 | cell-cell junction organization | 33 | 0.18 |
|  | GO:1990778 | protein localization to cell periphery | 46 | 0.14 |
|  | GO:0030198 | extracellular matrix organization | 48 | 0.13 |
|  | GO:0043062 | extracellular structure organization | 48 | 0.13 |
|  | GO:0001890 | placenta development | 27 | 0.18 |
|  | GO:0007173 | epidermal growth factor receptor signaling pathway | 23 | 0.19 |
| Brown  (558) | GO:0034470 | ncRNA processing | 45 | 0.12 |
|  | GO:0034660 | ncRNA metabolic process | 50 | 0.11 |
|  | GO:0042254 | ribosome biogenesis | 38 | 0.13 |
|  | GO:0022613 | ribonucleoprotein complex biogenesis | 47 | 0.1 |
|  | GO:0006364 | rRNA processing | 28 | 0.13 |
|  | GO:0016072 | rRNA metabolic process | 28 | 0.12 |
| Yellow  (447) | GO:0000280 | nuclear division | 66 | 0.16 |
|  | GO:0140014 | mitotic nuclear division | 55 | 0.21 |
|  | GO:0007059 | chromosome segregation | 59 | 0.18 |
|  | GO:0048285 | organelle fission | 67 | 0.15 |
|  | GO:0006260 | DNA replication | 54 | 0.2 |
|  | GO:0000070 | mitotic sister chromatid segregation | 41 | 0.27 |
| Green  (408) | GO:0045333 | cellular respiration | 35 | 0.18 |
|  | GO:0015980 | energy derivation by oxidation of organic compounds | 39 | 0.14 |
|  | GO:0006099 | tricarboxylic acid cycle | 16 | 0.47 |
|  | GO:0006119 | oxidative phosphorylation | 26 | 0.18 |
|  | GO:0009060 | aerobic respiration | 21 | 0.24 |
|  | GO:0022900 | electron transport chain | 25 | 0.13 |
| Red  (186) | GO:0045815 | positive regulation of gene expression, epigenetic | 5 | 0.09 |
|  | GO:0051099 | positive regulation of binding | 7 | 0.04 |
|  | GO:0031445 | regulation of heterochromatin assembly | 3 | 0.17 |
|  | GO:0010847 | regulation of chromatin assembly | 3 | 0.15 |
|  | GO:0001672 | regulation of chromatin assembly or disassembly | 3 | 0.12 |
|  | GO:0046856 | phosphatidylinositol dephosphorylation | 3 | 0.12 |
