# Supplementary material for: Identification of key genes and biological processes contributing to colitis associated dysplasia in ulcerative colitis
Source: PeerJ. 2021 Apr 27;9:e11321. doi: 10.7717/peerj.11321 (PMC8086577; doi:10.7717/peerj.11321)
Supplement: Supplemental Information 4 [file peerj-09-11321-s004.docx]

**Table S3. GSEA analysis of GO-BP terms between sporadic CRC and normal controls.**

| **ID** | **GO-BP terms** | **NES** | **P value** |
| --- | --- | --- | --- |
| GO:0034660 | ncRNA metabolic process | 2.379824 | 4.00E-27 |
| GO:0034470 | ncRNA processing | 2.417515 | 2.27E-25 |
| GO:0022613 | ribonucleoprotein complex biogenesis | 2.383617 | 9.16E-25 |
| GO:0042254 | ribosome biogenesis | 2.359761 | 3.11E-20 |
| GO:0006260 | DNA replication | 2.339368 | 9.26E-19 |
| GO:0000280 | nuclear division | 2.151342 | 6.16E-18 |
| GO:0071103 | DNA conformation change | 2.197858 | 1.02E-17 |
| GO:0042391 | regulation of membrane potential | -2.09793 | 2.96E-17 |
| GO:0006364 | rRNA processing | 2.379276 | 8.00E-17 |
| GO:0007059 | chromosome segregation | 2.15021 | 4.01E-15 |
| GO:0043588 | skin development | 2.032974 | 4.50E-15 |
| GO:0048285 | organelle fission | 1.99436 | 7.33E-15 |
| GO:0016072 | rRNA metabolic process | 2.252912 | 2.56E-14 |
| GO:0072503 | cellular divalent inorganic cation homeostasis | -1.95265 | 3.19E-14 |
| GO:0006261 | DNA-dependent DNA replication | 2.431238 | 6.06E-14 |
| GO:0008544 | epidermis development | 1.932365 | 2.32E-13 |
| GO:0098813 | nuclear chromosome segregation | 2.163242 | 3.40E-13 |
| GO:0001508 | action potential | -2.28696 | 1.02E-12 |
| GO:0000819 | sister chromatid segregation | 2.278586 | 1.33E-12 |
| GO:0006874 | cellular calcium ion homeostasis | -1.91985 | 1.63E-12 |
| GO:0003012 | muscle system process | -1.92026 | 3.14E-12 |
| GO:0019932 | second-messenger-mediated signaling | -1.91643 | 3.17E-12 |
| GO:0006302 | double-strand break repair | 2.096234 | 3.50E-12 |
| GO:0006310 | DNA recombination | 2.038026 | 4.03E-12 |
| GO:0140014 | mitotic nuclear division | 2.073518 | 4.45E-12 |
| GO:0051321 | meiotic cell cycle | 2.076595 | 9.65E-12 |
| GO:0055074 | calcium ion homeostasis | -1.86245 | 1.15E-11 |
| GO:0006399 | tRNA metabolic process | 2.222877 | 1.46E-11 |
| GO:0050804 | modulation of chemical synaptic transmission | -1.89262 | 1.55E-11 |
| GO:0030216 | keratinocyte differentiation | 1.970319 | 1.65E-11 |
| GO:1903046 | meiotic cell cycle process | 2.177063 | 2.18E-11 |
| GO:0099177 | regulation of trans-synaptic signaling | -1.89032 | 7.50E-11 |
| GO:0051480 | regulation of cytosolic calcium ion concentration | -1.92869 | 8.35E-11 |
| GO:0035637 | multicellular organismal signaling | -2.06198 | 1.34E-10 |
| GO:0000956 | nuclear-transcribed mRNA catabolic process | 2.06636 | 1.62E-10 |
| GO:0071824 | protein-DNA complex subunit organization | 1.968834 | 2.03E-10 |
| GO:0006323 | DNA packaging | 2.001819 | 2.83E-10 |
| GO:0009913 | epidermal cell differentiation | 1.871067 | 3.07E-10 |
| GO:0006936 | muscle contraction | -1.88063 | 3.16E-10 |
| GO:0000075 | cell cycle checkpoint | 2.051471 | 3.17E-10 |
| GO:0140013 | meiotic nuclear division | 2.13842 | 3.26E-10 |
| GO:0022618 | ribonucleoprotein complex assembly | 2.085957 | 3.50E-10 |
| GO:0034765 | regulation of ion transmembrane transport | -1.79268 | 3.81E-10 |
| GO:0071826 | ribonucleoprotein complex subunit organization | 2.065824 | 3.91E-10 |
| GO:0000070 | mitotic sister chromatid segregation | 2.200992 | 4.56E-10 |
| GO:0019080 | viral gene expression | 2.054626 | 4.97E-10 |
| GO:0007187 | G protein-coupled receptor signaling pathway, coupled to cyclic nucleotide second messenger | -1.98969 | 5.01E-10 |
| GO:0031424 | keratinization | 1.996309 | 7.00E-10 |
| GO:0051783 | regulation of nuclear division | 2.075199 | 8.63E-10 |
| GO:0000723 | telomere maintenance | 2.12205 | 1.02E-09 |
| GO:0031570 | DNA integrity checkpoint | 2.127235 | 2.19E-09 |
| GO:0000724 | double-strand break repair via homologous recombination | 2.164031 | 2.37E-09 |
| GO:0065004 | protein-DNA complex assembly | 1.945084 | 2.56E-09 |
| GO:0019935 | cyclic-nucleotide-mediated signaling | -1.98799 | 2.82E-09 |
| GO:0032200 | telomere organization | 2.096448 | 3.05E-09 |
| GO:0060271 | cilium assembly | 1.796468 | 3.69E-09 |
| GO:0090257 | regulation of muscle system process | -1.92629 | 3.76E-09 |
| GO:0050808 | synapse organization | -1.79777 | 4.72E-09 |
| GO:0034728 | nucleosome organization | 2.03392 | 4.91E-09 |
| GO:0019083 | viral transcription | 2.06747 | 6.37E-09 |
| GO:0000184 | nuclear-transcribed mRNA catabolic process, nonsense-mediated decay | 2.163603 | 6.61E-09 |
| GO:0006334 | nucleosome assembly | 2.105093 | 7.33E-09 |
| GO:0032392 | DNA geometric change | 2.178579 | 7.38E-09 |
| GO:0031497 | chromatin assembly | 1.959014 | 9.55E-09 |
| GO:0007188 | adenylate cyclase-modulating G protein-coupled receptor signaling pathway | -1.9539 | 9.74E-09 |
| GO:0000725 | recombinational repair | 2.173169 | 1.07E-08 |
| GO:0007204 | positive regulation of cytosolic calcium ion concentration | -1.84996 | 1.55E-08 |
| GO:0032508 | DNA duplex unwinding | 2.176344 | 1.64E-08 |
| GO:1903522 | regulation of blood circulation | -1.86264 | 1.66E-08 |
| GO:0022898 | regulation of transmembrane transporter activity | -1.87696 | 1.73E-08 |
| GO:0006401 | RNA catabolic process | 1.726151 | 2.25E-08 |
| GO:0001505 | regulation of neurotransmitter levels | -1.79501 | 2.49E-08 |
| GO:0008016 | regulation of heart contraction | -1.88867 | 2.68E-08 |
| GO:0071166 | ribonucleoprotein complex localization | 2.105491 | 2.82E-08 |
| GO:0006403 | RNA localization | 1.869109 | 2.99E-08 |
| GO:0006405 | RNA export from nucleus | 2.13142 | 3.17E-08 |
| GO:0010959 | regulation of metal ion transport | -1.75223 | 3.51E-08 |
| GO:0032412 | regulation of ion transmembrane transporter activity | -1.87941 | 3.62E-08 |
| GO:0032409 | regulation of transporter activity | -1.86778 | 3.69E-08 |
| GO:0008033 | tRNA processing | 2.149071 | 4.29E-08 |
| GO:0006333 | chromatin assembly or disassembly | 1.847617 | 5.00E-08 |
| GO:0090068 | positive regulation of cell cycle process | 1.79432 | 5.10E-08 |
| GO:0060047 | heart contraction | -1.81763 | 5.88E-08 |
| GO:0071426 | ribonucleoprotein complex export from nucleus | 2.104471 | 6.29E-08 |
| GO:0008217 | regulation of blood pressure | -1.93629 | 6.31E-08 |
| GO:0044782 | cilium organization | 1.693919 | 9.19E-08 |
| GO:0048167 | regulation of synaptic plasticity | -1.89661 | 9.75E-08 |
| GO:0007409 | axonogenesis | -1.67488 | 1.06E-07 |
| GO:0007093 | mitotic cell cycle checkpoint | 1.976801 | 1.20E-07 |
| GO:0006611 | protein export from nucleus | 1.913256 | 1.47E-07 |
| GO:0051168 | nuclear export | 1.882147 | 1.62E-07 |
| GO:0071867 | response to monoamine | -2.0196 | 1.65E-07 |
| GO:0071869 | response to catecholamine | -2.0196 | 1.65E-07 |
| GO:0006402 | mRNA catabolic process | 1.695345 | 1.76E-07 |
| GO:0006813 | potassium ion transport | -1.84301 | 2.02E-07 |
| GO:0003015 | heart process | -1.78146 | 2.15E-07 |
| GO:2001257 | regulation of cation channel activity | -1.89864 | 2.26E-07 |
| GO:0000077 | DNA damage checkpoint | 1.959874 | 2.61E-07 |
| GO:0006937 | regulation of muscle contraction | -1.92135 | 2.99E-07 |
| GO:0019933 | cAMP-mediated signaling | -1.85051 | 3.44E-07 |
| GO:0045787 | positive regulation of cell cycle | 1.66963 | 3.69E-07 |
| GO:0006814 | sodium ion transport | -1.84313 | 3.96E-07 |
| GO:0007088 | regulation of mitotic nuclear division | 1.930193 | 4.76E-07 |
| GO:1904062 | regulation of cation transmembrane transport | -1.74779 | 5.16E-07 |
| GO:0070838 | divalent metal ion transport | -1.63416 | 6.32E-07 |
| GO:0071868 | cellular response to monoamine stimulus | -1.96576 | 8.18E-07 |
| GO:0071870 | cellular response to catecholamine stimulus | -1.96576 | 8.18E-07 |
| GO:0061982 | meiosis I cell cycle process | 1.975944 | 9.40E-07 |
| GO:0071805 | potassium ion transmembrane transport | -1.80669 | 1.14E-06 |
| GO:0061337 | cardiac conduction | -1.89818 | 1.21E-06 |
| GO:0043270 | positive regulation of ion transport | -1.76001 | 1.26E-06 |
| GO:2001251 | negative regulation of chromosome organization | 1.909656 | 1.32E-06 |
| GO:0072331 | signal transduction by p53 class mediator | 1.696704 | 1.38E-06 |
| GO:0030258 | lipid modification | -1.76715 | 1.54E-06 |
| GO:0019722 | calcium-mediated signaling | -1.78086 | 1.58E-06 |
| GO:0050657 | nucleic acid transport | 1.793919 | 1.58E-06 |
| GO:0050658 | RNA transport | 1.793919 | 1.58E-06 |
| GO:0006816 | calcium ion transport | -1.63395 | 1.64E-06 |
| GO:0006836 | neurotransmitter transport | -1.71413 | 1.68E-06 |
| GO:0070268 | cornification | 1.983721 | 1.72E-06 |
| GO:0034440 | lipid oxidation | -1.95465 | 1.76E-06 |
| GO:0090305 | nucleic acid phosphodiester bond hydrolysis | 1.67553 | 1.78E-06 |
| GO:0061458 | reproductive system development | 1.539165 | 1.94E-06 |
| GO:0072511 | divalent inorganic cation transport | -1.578 | 1.95E-06 |
| GO:0031047 | gene silencing by RNA | 1.812699 | 2.08E-06 |
| GO:0051236 | establishment of RNA localization | 1.784321 | 2.30E-06 |
| GO:0006821 | chloride transport | -1.93989 | 2.34E-06 |
| GO:0090066 | regulation of anatomical structure size | -1.57126 | 2.39E-06 |
| GO:0060078 | regulation of postsynaptic membrane potential | -1.86509 | 2.51E-06 |
| GO:0007269 | neurotransmitter secretion | -1.80335 | 2.56E-06 |
| GO:0099643 | signal release from synapse | -1.80335 | 2.56E-06 |
| GO:0031023 | microtubule organizing center organization | 1.891334 | 2.63E-06 |
| GO:1901987 | regulation of cell cycle phase transition | 1.555465 | 3.06E-06 |
| GO:0040029 | regulation of gene expression, epigenetic | 1.642807 | 3.31E-06 |
| GO:0042770 | signal transduction in response to DNA damage | 1.886798 | 3.50E-06 |
| GO:0006275 | regulation of DNA replication | 1.964887 | 4.18E-06 |
| GO:0050806 | positive regulation of synaptic transmission | -1.77737 | 4.40E-06 |
| GO:0072599 | establishment of protein localization to endoplasmic reticulum | 1.953917 | 4.81E-06 |
| GO:0044774 | mitotic DNA integrity checkpoint | 1.962099 | 5.04E-06 |
| GO:0045047 | protein targeting to ER | 1.952012 | 5.16E-06 |
| GO:0006413 | translational initiation | 1.759281 | 5.18E-06 |
| GO:1901990 | regulation of mitotic cell cycle phase transition | 1.563068 | 5.24E-06 |
| GO:0032411 | positive regulation of transporter activity | -1.89645 | 5.25E-06 |
| GO:0007051 | spindle organization | 1.775758 | 5.45E-06 |
| GO:0048608 | reproductive structure development | 1.539289 | 5.60E-06 |
| GO:0044839 | cell cycle G2/M phase transition | 1.667269 | 5.94E-06 |
| GO:0006406 | mRNA export from nucleus | 1.920004 | 6.23E-06 |
| GO:0071427 | mRNA-containing ribonucleoprotein complex export from nucleus | 1.920004 | 6.23E-06 |
| GO:0099003 | vesicle-mediated transport in synapse | -1.74163 | 6.48E-06 |
| GO:0051028 | mRNA transport | 1.864619 | 6.62E-06 |
| GO:1902850 | microtubule cytoskeleton organization involved in mitosis | 1.893157 | 8.04E-06 |
| GO:0031123 | RNA 3'-end processing | 1.850344 | 8.42E-06 |
| GO:0016458 | gene silencing | 1.67211 | 9.24E-06 |
| GO:0006338 | chromatin remodeling | 1.71432 | 9.92E-06 |
| GO:1902749 | regulation of cell cycle G2/M phase transition | 1.668861 | 1.27E-05 |
| GO:0051051 | negative regulation of transport | -1.53702 | 1.30E-05 |
| GO:0035194 | post-transcriptional gene silencing by RNA | 1.776052 | 1.45E-05 |
| GO:0007626 | locomotory behavior | -1.73794 | 1.48E-05 |
| GO:0030330 | DNA damage response, signal transduction by p53 class mediator | 1.841174 | 1.55E-05 |
| GO:0032543 | mitochondrial translation | 1.819607 | 1.55E-05 |
| GO:0016441 | posttranscriptional gene silencing | 1.770671 | 1.67E-05 |
| GO:0045666 | positive regulation of neuron differentiation | -1.60112 | 1.75E-05 |
| GO:0051052 | regulation of DNA metabolic process | 1.568823 | 1.75E-05 |
| GO:0007098 | centrosome cycle | 1.842515 | 1.76E-05 |
| GO:0007127 | meiosis I | 1.85876 | 1.89E-05 |
| GO:0099504 | synaptic vesicle cycle | -1.70935 | 1.92E-05 |
| GO:1901796 | regulation of signal transduction by p53 class mediator | 1.733816 | 2.01E-05 |
| GO:0000082 | G1/S transition of mitotic cell cycle | 1.639077 | 2.02E-05 |
| GO:0048568 | embryonic organ development | 1.484549 | 2.15E-05 |
| GO:0042445 | hormone metabolic process | -1.69859 | 2.28E-05 |
| GO:0050890 | cognition | -1.61854 | 2.62E-05 |
| GO:0140053 | mitochondrial gene expression | 1.752354 | 2.71E-05 |
| GO:0051054 | positive regulation of DNA metabolic process | 1.654489 | 2.77E-05 |
| GO:0009410 | response to xenobiotic stimulus | -1.61962 | 2.91E-05 |
| GO:0098742 | cell-cell adhesion via plasma-membrane adhesion molecules | -1.63433 | 2.94E-05 |
| GO:0032414 | positive regulation of ion transmembrane transporter activity | -1.84038 | 3.17E-05 |
| GO:0042303 | molting cycle | 1.8328 | 3.55E-05 |
| GO:0042633 | hair cycle | 1.8328 | 3.55E-05 |
| GO:0016042 | lipid catabolic process | -1.58943 | 3.76E-05 |
| GO:0071248 | cellular response to metal ion | -1.67215 | 3.97E-05 |
| GO:0060968 | regulation of gene silencing | 1.754258 | 4.03E-05 |
| GO:0000086 | G2/M transition of mitotic cell cycle | 1.607011 | 4.16E-05 |
| GO:0032963 | collagen metabolic process | 1.844928 | 4.17E-05 |
| GO:0060401 | cytosolic calcium ion transport | -1.69245 | 4.56E-05 |
| GO:0006289 | nucleotide-excision repair | 1.837025 | 4.79E-05 |
| GO:0002250 | adaptive immune response | -1.55557 | 4.87E-05 |
| GO:0051588 | regulation of neurotransmitter transport | -1.73784 | 4.89E-05 |
| GO:0071466 | cellular response to xenobiotic stimulus | -1.69757 | 5.04E-05 |
| GO:0003002 | regionalization | 1.494928 | 5.20E-05 |
| GO:0006939 | smooth muscle contraction | -1.80176 | 5.44E-05 |
| GO:0007189 | adenylate cyclase-activating G protein-coupled receptor signaling pathway | -1.7581 | 5.61E-05 |
| GO:0002764 | immune response-regulating signaling pathway | -1.53904 | 5.72E-05 |
| GO:0070588 | calcium ion transmembrane transport | -1.58972 | 5.73E-05 |
| GO:0090501 | RNA phosphodiester bond hydrolysis | 1.769985 | 5.77E-05 |
| GO:0010976 | positive regulation of neuron projection development | -1.60473 | 5.83E-05 |
| GO:0031346 | positive regulation of cell projection organization | -1.55189 | 6.06E-05 |
| GO:0044843 | cell cycle G1/S phase transition | 1.571241 | 6.28E-05 |
| GO:0090596 | sensory organ morphogenesis | 1.579985 | 6.71E-05 |
| GO:0034764 | positive regulation of transmembrane transport | -1.64612 | 6.81E-05 |
| GO:0007389 | pattern specification process | 1.442639 | 6.92E-05 |
| GO:0035195 | gene silencing by miRNA | 1.726817 | 6.99E-05 |
| GO:2000241 | regulation of reproductive process | 1.70505 | 7.05E-05 |
| GO:0003018 | vascular process in circulatory system | -1.67859 | 7.56E-05 |
| GO:0010975 | regulation of neuron projection development | -1.49823 | 7.65E-05 |
| GO:0030198 | extracellular matrix organization | 1.464412 | 7.76E-05 |
| GO:0015711 | organic anion transport | -1.47961 | 7.78E-05 |
| GO:0097305 | response to alcohol | -1.64209 | 8.13E-05 |
| GO:0010389 | regulation of G2/M transition of mitotic cell cycle | 1.600111 | 8.25E-05 |
| GO:0072659 | protein localization to plasma membrane | -1.58885 | 8.32E-05 |
| GO:0035296 | regulation of tube diameter | -1.71085 | 8.40E-05 |
| GO:0097746 | regulation of blood vessel diameter | -1.71085 | 8.40E-05 |
| GO:1903305 | regulation of regulated secretory pathway | -1.70039 | 8.47E-05 |
| GO:0016051 | carbohydrate biosynthetic process | -1.66684 | 8.72E-05 |
| GO:0019730 | antimicrobial humoral response | 1.764584 | 9.19E-05 |
| GO:0007411 | axon guidance | -1.56972 | 9.30E-05 |
| GO:0034754 | cellular hormone metabolic process | -1.7374 | 9.49E-05 |
| GO:0002768 | immune response-regulating cell surface receptor signaling pathway | -1.54666 | 9.59E-05 |
| GO:1904950 | negative regulation of establishment of protein localization | -1.6311 | 0.000104 |
| GO:0035150 | regulation of tube size | -1.71167 | 0.000105 |
| GO:0006941 | striated muscle contraction | -1.67006 | 0.000109 |
| GO:0007611 | learning or memory | -1.60572 | 0.000109 |
| GO:0007052 | mitotic spindle organization | 1.777739 | 0.00012 |
| GO:0035690 | cellular response to drug | -1.47826 | 0.000131 |
| GO:1905330 | regulation of morphogenesis of an epithelium | 1.620971 | 0.000134 |
| GO:0006066 | alcohol metabolic process | -1.52777 | 0.000135 |
| GO:0006805 | xenobiotic metabolic process | -1.72445 | 0.000136 |
| GO:0072676 | lymphocyte migration | -1.73551 | 0.000136 |
| GO:1902903 | regulation of supramolecular fiber organization | -1.53036 | 0.000141 |
| GO:0043062 | extracellular structure organization | 1.461216 | 0.000145 |
| GO:0097485 | neuron projection guidance | -1.56719 | 0.000155 |
| GO:0048732 | gland development | 1.416074 | 0.00016 |
| GO:0032970 | regulation of actin filament-based process | -1.511 | 0.000172 |
| GO:0098693 | regulation of synaptic vesicle cycle | -1.74387 | 0.000177 |
| GO:0048705 | skeletal system morphogenesis | 1.54703 | 0.000178 |
| GO:0060147 | regulation of posttranscriptional gene silencing | 1.755883 | 0.000179 |
| GO:0060966 | regulation of gene silencing by RNA | 1.755883 | 0.000179 |
| GO:0070372 | regulation of ERK1 and ERK2 cascade | -1.55811 | 0.000181 |
| GO:0001890 | placenta development | 1.638879 | 0.000182 |
| GO:0032956 | regulation of actin cytoskeleton organization | -1.51391 | 0.000185 |
| GO:0016079 | synaptic vesicle exocytosis | -1.7181 | 0.000185 |
| GO:0060402 | calcium ion transport into cytosol | -1.6544 | 0.000185 |
| GO:0016331 | morphogenesis of embryonic epithelium | 1.630129 | 0.000189 |
| GO:0035725 | sodium ion transmembrane transport | -1.68404 | 0.000204 |
| GO:0099565 | chemical synaptic transmission, postsynaptic | -1.74826 | 0.000209 |
| GO:0051235 | maintenance of location | -1.51525 | 0.000216 |
| GO:0007416 | synapse assembly | -1.63659 | 0.000217 |
| GO:0050796 | regulation of insulin secretion | -1.63654 | 0.000217 |
| GO:0002685 | regulation of leukocyte migration | -1.63505 | 0.000223 |
| GO:0015850 | organic hydroxy compound transport | -1.57595 | 0.000225 |
| GO:0070661 | leukocyte proliferation | -1.54578 | 0.000226 |
| GO:0060048 | cardiac muscle contraction | -1.69095 | 0.000228 |
| GO:0009952 | anterior/posterior pattern specification | 1.531161 | 0.000234 |
| GO:1990778 | protein localization to cell periphery | -1.53359 | 0.000245 |
| GO:0008380 | RNA splicing | 1.370414 | 0.000249 |
| GO:0017157 | regulation of exocytosis | -1.609 | 0.000256 |
| GO:0042110 | T cell activation | -1.43516 | 0.000273 |
| GO:0060964 | regulation of gene silencing by miRNA | 1.711424 | 0.000274 |
| GO:0015698 | inorganic anion transport | -1.63392 | 0.000276 |
| GO:0051224 | negative regulation of protein transport | -1.59818 | 0.000282 |
| GO:0044242 | cellular lipid catabolic process | -1.57079 | 0.000288 |
| GO:0002694 | regulation of leukocyte activation | -1.43485 | 0.000288 |
| GO:2000027 | regulation of animal organ morphogenesis | 1.501823 | 0.000299 |
| GO:0007159 | leukocyte cell-cell adhesion | -1.52411 | 0.000316 |
| GO:0046928 | regulation of neurotransmitter secretion | -1.72455 | 0.000329 |
| GO:0009062 | fatty acid catabolic process | -1.72867 | 0.00034 |
| GO:0043414 | macromolecule methylation | 1.447111 | 0.00035 |
| GO:0030073 | insulin secretion | -1.59996 | 0.000355 |
| GO:0034329 | cell junction assembly | -1.48243 | 0.000365 |
| GO:0070371 | ERK1 and ERK2 cascade | -1.50822 | 0.000394 |
| GO:0009451 | RNA modification | 1.632044 | 0.000414 |
| GO:1902904 | negative regulation of supramolecular fiber organization | -1.65837 | 0.000417 |
| GO:0006397 | mRNA processing | 1.344057 | 0.000417 |
| GO:0051924 | regulation of calcium ion transport | -1.53926 | 0.000426 |
| GO:0001764 | neuron migration | -1.62169 | 0.00043 |
| GO:0040013 | negative regulation of locomotion | -1.48977 | 0.000466 |
| GO:0010038 | response to metal ion | -1.47599 | 0.000467 |
| GO:0045930 | negative regulation of mitotic cell cycle | 1.435972 | 0.000476 |
| GO:0006415 | translational termination | 1.660223 | 0.000479 |
| GO:0000375 | RNA splicing, via transesterification reactions | 1.412888 | 0.000487 |
| GO:0006913 | nucleocytoplasmic transport | 1.412681 | 0.000487 |
| GO:0048017 | inositol lipid-mediated signaling | -1.5945 | 0.000492 |
| GO:1903531 | negative regulation of secretion by cell | -1.56751 | 0.000496 |
| GO:0098656 | anion transmembrane transport | -1.49993 | 0.000514 |
| GO:0000377 | RNA splicing, via transesterification reactions with bulged adenosine as nucleophile | 1.406409 | 0.000522 |
| GO:0000398 | mRNA splicing, via spliceosome | 1.406409 | 0.000522 |
| GO:0051271 | negative regulation of cellular component movement | -1.4842 | 0.000525 |
| GO:0015837 | amine transport | -1.67397 | 0.000526 |
| GO:0022604 | regulation of cell morphogenesis | -1.40587 | 0.000543 |
| GO:0071241 | cellular response to inorganic substance | -1.53991 | 0.000561 |
| GO:0048592 | eye morphogenesis | 1.6177 | 0.000562 |
| GO:0055067 | monovalent inorganic cation homeostasis | -1.60065 | 0.000575 |
| GO:0002429 | immune response-activating cell surface receptor signaling pathway | -1.46099 | 0.000589 |
| GO:0002757 | immune response-activating signal transduction | -1.46099 | 0.000589 |
| GO:0007215 | glutamate receptor signaling pathway | -1.65747 | 0.000663 |
| GO:0001704 | formation of primary germ layer | 1.62555 | 0.000667 |
| GO:0051169 | nuclear transport | 1.423748 | 0.000672 |
| GO:0048659 | smooth muscle cell proliferation | -1.63493 | 0.000674 |
| GO:0051048 | negative regulation of secretion | -1.56356 | 0.000675 |
| GO:0050900 | leukocyte migration | -1.43082 | 0.0007 |
| GO:0016358 | dendrite development | -1.52131 | 0.000709 |
| GO:0016197 | endosomal transport | -1.54548 | 0.000737 |
| GO:0050769 | positive regulation of neurogenesis | -1.43222 | 0.000774 |
| GO:0002253 | activation of immune response | -1.43701 | 0.000781 |
| GO:0007015 | actin filament organization | -1.46024 | 0.000784 |
| GO:0015931 | nucleobase-containing compound transport | 1.475148 | 0.000808 |
| GO:0006631 | fatty acid metabolic process | -1.46852 | 0.000855 |
| GO:0048660 | regulation of smooth muscle cell proliferation | -1.62588 | 0.000888 |
| GO:0019233 | sensory perception of pain | -1.67416 | 0.000897 |
| GO:0030100 | regulation of endocytosis | -1.5161 | 0.000908 |
| GO:0070663 | regulation of leukocyte proliferation | -1.52281 | 0.000921 |
| GO:0048015 | phosphatidylinositol-mediated signaling | -1.56854 | 0.00095 |
| GO:0098661 | inorganic anion transmembrane transport | -1.67187 | 0.000954 |
| GO:0032102 | negative regulation of response to external stimulus | -1.44776 | 0.000993 |
| GO:0060349 | bone morphogenesis | 1.627219 | 0.001015 |
| GO:0033044 | regulation of chromosome organization | 1.397295 | 0.001047 |
| GO:0007156 | homophilic cell adhesion via plasma membrane adhesion molecules | -1.53132 | 0.001048 |
| GO:0048562 | embryonic organ morphogenesis | 1.410448 | 0.001054 |
| GO:0031348 | negative regulation of defense response | -1.53373 | 0.001076 |
| GO:1903509 | liposaccharide metabolic process | -1.62271 | 0.001094 |
| GO:0042063 | gliogenesis | -1.45731 | 0.00111 |
| GO:0070374 | positive regulation of ERK1 and ERK2 cascade | -1.51844 | 0.00113 |
| GO:0030336 | negative regulation of cell migration | -1.48397 | 0.001156 |
| GO:0048593 | camera-type eye morphogenesis | 1.61709 | 0.00119 |
| GO:0021700 | developmental maturation | -1.46865 | 0.001203 |
| GO:0051963 | regulation of synapse assembly | -1.57854 | 0.00123 |
| GO:0002792 | negative regulation of peptide secretion | -1.6022 | 0.001233 |
| GO:0010948 | negative regulation of cell cycle process | 1.40232 | 0.001237 |
| GO:0034767 | positive regulation of ion transmembrane transport | -1.57606 | 0.001259 |
| GO:0050920 | regulation of chemotaxis | -1.50899 | 0.001259 |
| GO:0110053 | regulation of actin filament organization | -1.48571 | 0.001306 |
| GO:2000146 | negative regulation of cell motility | -1.48126 | 0.001323 |
| GO:0072329 | monocarboxylic acid catabolic process | -1.60934 | 0.00135 |
| GO:1901653 | cellular response to peptide | -1.42368 | 0.001365 |
| GO:0071897 | DNA biosynthetic process | 1.489036 | 0.0014 |
| GO:0003014 | renal system process | -1.609 | 0.001419 |
| GO:0043500 | muscle adaptation | -1.64677 | 0.001428 |
| GO:0007612 | learning | -1.54101 | 0.001459 |
| GO:0051282 | regulation of sequestering of calcium ion | -1.5807 | 0.001482 |
| GO:1901991 | negative regulation of mitotic cell cycle phase transition | 1.416601 | 0.001483 |
| GO:0007162 | negative regulation of cell adhesion | -1.4752 | 0.001529 |
| GO:0046651 | lymphocyte proliferation | -1.47206 | 0.001542 |
| GO:1901888 | regulation of cell junction assembly | -1.492 | 0.001589 |
| GO:2001020 | regulation of response to DNA damage stimulus | 1.448367 | 0.001592 |
| GO:0050709 | negative regulation of protein secretion | -1.58723 | 0.001687 |
| GO:0071383 | cellular response to steroid hormone stimulus | -1.4525 | 0.001763 |
| GO:0046942 | carboxylic acid transport | -1.42108 | 0.001784 |
| GO:0006354 | DNA-templated transcription, elongation | 1.589008 | 0.001819 |
| GO:0050863 | regulation of T cell activation | -1.45767 | 0.001857 |
| GO:0051209 | release of sequestered calcium ion into cytosol | -1.57196 | 0.001918 |
| GO:0014065 | phosphatidylinositol 3-kinase signaling | -1.60852 | 0.001931 |
| GO:0014066 | regulation of phosphatidylinositol 3-kinase signaling | -1.57645 | 0.001932 |
| GO:0098754 | detoxification | -1.55847 | 0.001971 |
| GO:0032259 | methylation | 1.353772 | 0.002016 |
| GO:0046677 | response to antibiotic | -1.47836 | 0.002035 |
| GO:0032943 | mononuclear cell proliferation | -1.47762 | 0.002043 |
| GO:0006664 | glycolipid metabolic process | -1.62442 | 0.002054 |
| GO:0001558 | regulation of cell growth | -1.38431 | 0.002071 |
| GO:1902532 | negative regulation of intracellular signal transduction | -1.36789 | 0.002073 |
| GO:0048545 | response to steroid hormone | -1.40136 | 0.002081 |
| GO:0051225 | spindle assembly | 1.556288 | 0.002262 |
| GO:0051249 | regulation of lymphocyte activation | -1.41336 | 0.002266 |
| GO:0050728 | negative regulation of inflammatory response | -1.55553 | 0.00227 |
| GO:0051283 | negative regulation of sequestering of calcium ion | -1.56739 | 0.0024 |
| GO:0044262 | cellular carbohydrate metabolic process | -1.45566 | 0.002445 |
| GO:0050851 | antigen receptor-mediated signaling pathway | -1.49969 | 0.002472 |
| GO:0051208 | sequestering of calcium ion | -1.56017 | 0.002524 |
| GO:0042476 | odontogenesis | 1.518356 | 0.002545 |
| GO:1904064 | positive regulation of cation transmembrane transport | -1.53612 | 0.002688 |
| GO:0030879 | mammary gland development | 1.495351 | 0.0027 |
| GO:1903037 | regulation of leukocyte cell-cell adhesion | -1.45243 | 0.002703 |
| GO:0016049 | cell growth | -1.34921 | 0.002723 |
| GO:0018108 | peptidyl-tyrosine phosphorylation | -1.40213 | 0.002727 |
| GO:0051651 | maintenance of location in cell | -1.48402 | 0.002769 |
| GO:1902750 | negative regulation of cell cycle G2/M phase transition | 1.556142 | 0.002837 |
| GO:0032944 | regulation of mononuclear cell proliferation | -1.47027 | 0.003003 |
| GO:0015849 | organic acid transport | -1.43446 | 0.00314 |
| GO:0051017 | actin filament bundle assembly | -1.5344 | 0.003141 |
| GO:1901988 | negative regulation of cell cycle phase transition | 1.379794 | 0.003198 |
| GO:0007292 | female gamete generation | 1.550092 | 0.00322 |
| GO:0043491 | protein kinase B signaling | -1.42434 | 0.003288 |
| GO:0018212 | peptidyl-tyrosine modification | -1.39908 | 0.003309 |
| GO:0061572 | actin filament bundle organization | -1.55212 | 0.003412 |
| GO:0022408 | negative regulation of cell-cell adhesion | -1.46517 | 0.003417 |
| GO:0030534 | adult behavior | -1.54759 | 0.003467 |
| GO:0055076 | transition metal ion homeostasis | -1.52383 | 0.003484 |
| GO:0007586 | digestion | -1.51288 | 0.003489 |
| GO:0048706 | embryonic skeletal system development | 1.547208 | 0.003557 |
| GO:0019751 | polyol metabolic process | -1.53168 | 0.003932 |
| GO:0060562 | epithelial tube morphogenesis | 1.328629 | 0.003948 |
| GO:0007369 | gastrulation | 1.474619 | 0.004024 |
| GO:0008202 | steroid metabolic process | -1.36851 | 0.004088 |
| GO:0032271 | regulation of protein polymerization | -1.44507 | 0.004104 |
| GO:0006282 | regulation of DNA repair | 1.52883 | 0.004157 |
| GO:0099173 | postsynapse organization | -1.50357 | 0.004219 |
| GO:0021915 | neural tube development | 1.488176 | 0.004225 |
| GO:0010001 | glial cell differentiation | -1.43596 | 0.004252 |
| GO:0042552 | myelination | -1.50276 | 0.004328 |
| GO:0010721 | negative regulation of cell development | -1.38145 | 0.004387 |
| GO:0045926 | negative regulation of growth | -1.41688 | 0.004458 |
| GO:0060541 | respiratory system development | 1.398689 | 0.004732 |
| GO:0007272 | ensheathment of neurons | -1.52496 | 0.004775 |
| GO:0008366 | axon ensheathment | -1.52496 | 0.004775 |
| GO:0062013 | positive regulation of small molecule metabolic process | -1.52448 | 0.004775 |
| GO:0032535 | regulation of cellular component size | -1.35341 | 0.004954 |
| GO:0010212 | response to ionizing radiation | 1.471065 | 0.005074 |
| GO:0002683 | negative regulation of immune system process | -1.34792 | 0.005086 |
| GO:0050670 | regulation of lymphocyte proliferation | -1.45923 | 0.005222 |
| GO:0051216 | cartilage development | 1.382229 | 0.005236 |
| GO:0030111 | regulation of Wnt signaling pathway | 1.293901 | 0.005237 |
| GO:0051056 | regulation of small GTPase mediated signal transduction | -1.38194 | 0.005305 |
| GO:0009101 | glycoprotein biosynthetic process | -1.35802 | 0.005346 |
| GO:0051494 | negative regulation of cytoskeleton organization | -1.48736 | 0.005362 |
| GO:0050708 | regulation of protein secretion | -1.34485 | 0.005404 |
| GO:0050803 | regulation of synapse structure or activity | -1.45923 | 0.005476 |
| GO:1903169 | regulation of calcium ion transmembrane transport | -1.4889 | 0.005681 |
| GO:0042471 | ear morphogenesis | 1.478647 | 0.005731 |
| GO:0031669 | cellular response to nutrient levels | -1.41275 | 0.005756 |
| GO:0098781 | ncRNA transcription | 1.507643 | 0.005792 |
| GO:0045471 | response to ethanol | -1.464 | 0.005867 |
| GO:0000018 | regulation of DNA recombination | 1.540199 | 0.005889 |
| GO:0008154 | actin polymerization or depolymerization | -1.45697 | 0.005893 |
| GO:0051961 | negative regulation of nervous system development | -1.36635 | 0.006018 |
| GO:0048736 | appendage development | 1.442947 | 0.006051 |
| GO:0060173 | limb development | 1.442947 | 0.006051 |
| GO:0050921 | positive regulation of chemotaxis | -1.48563 | 0.006098 |
| GO:0022407 | regulation of cell-cell adhesion | -1.36014 | 0.006196 |
| GO:0030004 | cellular monovalent inorganic cation homeostasis | -1.50599 | 0.006275 |
| GO:0007565 | female pregnancy | 1.398552 | 0.00629 |
| GO:0030183 | B cell differentiation | -1.47196 | 0.006359 |
| GO:0050807 | regulation of synapse organization | -1.42069 | 0.00647 |
| GO:1990845 | adaptive thermogenesis | -1.44358 | 0.006729 |
| GO:0097553 | calcium ion transmembrane import into cytosol | -1.49676 | 0.006817 |
| GO:0030335 | positive regulation of cell migration | -1.32638 | 0.006853 |
| GO:0006644 | phospholipid metabolic process | -1.34333 | 0.006967 |
| GO:0001523 | retinoid metabolic process | -1.45476 | 0.007039 |
| GO:0061008 | hepaticobiliary system development | 1.416624 | 0.007188 |
| GO:0031644 | regulation of nervous system process | -1.47302 | 0.007225 |
| GO:0030072 | peptide hormone secretion | -1.41037 | 0.007265 |
| GO:0007613 | memory | -1.49172 | 0.007353 |
| GO:0030326 | embryonic limb morphogenesis | 1.487608 | 0.007444 |
| GO:0035113 | embryonic appendage morphogenesis | 1.487608 | 0.007444 |
| GO:0097549 | chromatin organization involved in negative regulation of transcription | 1.488184 | 0.007455 |
| GO:1901654 | response to ketone | -1.44208 | 0.007469 |
| GO:0002221 | pattern recognition receptor signaling pathway | -1.40313 | 0.007912 |
| GO:0002687 | positive regulation of leukocyte migration | -1.44058 | 0.007961 |
| GO:0015718 | monocarboxylic acid transport | -1.4474 | 0.00805 |
| GO:0010951 | negative regulation of endopeptidase activity | 1.332221 | 0.008117 |
| GO:0042692 | muscle cell differentiation | -1.3649 | 0.008161 |
| GO:0008360 | regulation of cell shape | -1.40682 | 0.00824 |
| GO:0008406 | gonad development | 1.325885 | 0.008268 |
| GO:0016482 | cytosolic transport | -1.44529 | 0.008284 |
| GO:0071375 | cellular response to peptide hormone stimulus | -1.35417 | 0.008311 |
| GO:0001841 | neural tube formation | 1.446132 | 0.008622 |
| GO:0030098 | lymphocyte differentiation | -1.324 | 0.008724 |
| GO:0030048 | actin filament-based movement | -1.43947 | 0.008804 |
| GO:0007009 | plasma membrane organization | -1.47828 | 0.008853 |
| GO:0045137 | development of primary sexual characteristics | 1.322869 | 0.008891 |
| GO:0034401 | chromatin organization involved in regulation of transcription | 1.466787 | 0.008896 |
| GO:0051260 | protein homooligomerization | -1.3227 | 0.0089 |
| GO:0007548 | sex differentiation | 1.304547 | 0.009034 |
| GO:1902905 | positive regulation of supramolecular fiber organization | -1.38811 | 0.009055 |
| GO:0002526 | acute inflammatory response | 1.469954 | 0.009132 |
| GO:0046434 | organophosphate catabolic process | -1.4417 | 0.009207 |
| GO:0031668 | cellular response to extracellular stimulus | -1.35943 | 0.009332 |
| GO:0034504 | protein localization to nucleus | 1.324291 | 0.009462 |
| GO:0006721 | terpenoid metabolic process | -1.48427 | 0.009504 |
| GO:0046916 | cellular transition metal ion homeostasis | -1.48948 | 0.009521 |
| GO:0016101 | diterpenoid metabolic process | -1.45947 | 0.009648 |
| GO:0044282 | small molecule catabolic process | -1.30114 | 0.009706 |
| GO:0050770 | regulation of axonogenesis | -1.42104 | 0.009726 |
| GO:0106106 | cold-induced thermogenesis | -1.48709 | 0.009894 |
| GO:0120161 | regulation of cold-induced thermogenesis | -1.48709 | 0.009894 |
| GO:0035148 | tube formation | 1.418636 | 0.010089 |
| GO:0070972 | protein localization to endoplasmic reticulum | 1.467574 | 0.010129 |
| GO:1903038 | negative regulation of leukocyte cell-cell adhesion | -1.47347 | 0.010274 |
| GO:0042113 | B cell activation | -1.37627 | 0.010303 |
| GO:0006417 | regulation of translation | 1.27009 | 0.010483 |
| GO:0001701 | in utero embryonic development | 1.241665 | 0.01049 |
| GO:0006909 | phagocytosis | -1.39361 | 0.010542 |
| GO:0072175 | epithelial tube formation | 1.394963 | 0.010803 |
| GO:0071322 | cellular response to carbohydrate stimulus | -1.42692 | 0.010914 |
| GO:0001837 | epithelial to mesenchymal transition | 1.448164 | 0.010933 |
| GO:0060326 | cell chemotaxis | -1.34354 | 0.011105 |
| GO:0044264 | cellular polysaccharide metabolic process | -1.50168 | 0.011144 |
| GO:0051928 | positive regulation of calcium ion transport | -1.46264 | 0.011153 |
| GO:0042133 | neurotransmitter metabolic process | -1.43461 | 0.011191 |
| GO:0008037 | cell recognition | -1.44033 | 0.011469 |
| GO:0050773 | regulation of dendrite development | -1.43622 | 0.011469 |
| GO:0050906 | detection of stimulus involved in sensory perception | 1.227459 | 0.01161 |
| GO:0001838 | embryonic epithelial tube formation | 1.448269 | 0.011714 |
| GO:0001933 | negative regulation of protein phosphorylation | -1.3037 | 0.012109 |
| GO:0050768 | negative regulation of neurogenesis | -1.37316 | 0.012135 |
| GO:0005976 | polysaccharide metabolic process | -1.45231 | 0.012213 |
| GO:0008064 | regulation of actin polymerization or depolymerization | -1.41128 | 0.012229 |
| GO:0001889 | liver development | 1.435331 | 0.012354 |
| GO:0010466 | negative regulation of peptidase activity | 1.299617 | 0.012358 |
| GO:0019216 | regulation of lipid metabolic process | -1.29929 | 0.012453 |
| GO:0046683 | response to organophosphorus | -1.41374 | 0.012613 |
| GO:0046165 | alcohol biosynthetic process | -1.41976 | 0.012667 |
| GO:0001101 | response to acid chemical | -1.3106 | 0.012734 |
| GO:0002791 | regulation of peptide secretion | -1.28047 | 0.012744 |
| GO:0010876 | lipid localization | -1.3071 | 0.012797 |
| GO:0046486 | glycerolipid metabolic process | -1.31356 | 0.012856 |
| GO:0006869 | lipid transport | -1.31079 | 0.012953 |
| GO:0001763 | morphogenesis of a branching structure | 1.298575 | 0.013033 |
| GO:0009100 | glycoprotein metabolic process | -1.31715 | 0.013312 |
| GO:0031032 | actomyosin structure organization | -1.40931 | 0.013498 |
| GO:0071496 | cellular response to external stimulus | -1.30654 | 0.013595 |
| GO:0044706 | multi-multicellular organism process | 1.333724 | 0.01362 |
| GO:0010469 | regulation of signaling receptor activity | -1.40538 | 0.013798 |
| GO:0014074 | response to purine-containing compound | -1.42002 | 0.014108 |
| GO:0006720 | isoprenoid metabolic process | -1.41338 | 0.01429 |
| GO:0061448 | connective tissue development | 1.276512 | 0.014385 |
| GO:0051896 | regulation of protein kinase B signaling | -1.37218 | 0.014651 |
| GO:0070252 | actin-mediated cell contraction | -1.44729 | 0.01466 |
| GO:0048588 | developmental cell growth | -1.40161 | 0.015029 |
| GO:0051302 | regulation of cell division | 1.359751 | 0.015211 |
| GO:0045931 | positive regulation of mitotic cell cycle | 1.38427 | 0.015687 |
| GO:0060348 | bone development | 1.311277 | 0.015931 |
| GO:0010769 | regulation of cell morphogenesis involved in differentiation | -1.334 | 0.016352 |
| GO:0002062 | chondrocyte differentiation | 1.401832 | 0.016452 |
| GO:0045665 | negative regulation of neuron differentiation | -1.38471 | 0.016487 |
| GO:0030178 | negative regulation of Wnt signaling pathway | 1.292019 | 0.017485 |
| GO:0031099 | regeneration | 1.322575 | 0.017745 |
| GO:0001655 | urogenital system development | 1.226843 | 0.018459 |
| GO:1901342 | regulation of vasculature development | -1.30614 | 0.019909 |
| GO:0033002 | muscle cell proliferation | -1.37468 | 0.020088 |
| GO:0030832 | regulation of actin filament length | -1.36628 | 0.020353 |
| GO:0055001 | muscle cell development | -1.3819 | 0.02038 |
| GO:0003007 | heart morphogenesis | 1.274639 | 0.020395 |
| GO:0007519 | skeletal muscle tissue development | -1.39 | 0.020492 |
| GO:0090101 | negative regulation of transmembrane receptor protein serine/threonine kinase signaling pathway | -1.43372 | 0.020517 |
| GO:0001952 | regulation of cell-matrix adhesion | -1.41799 | 0.020887 |
| GO:0002224 | toll-like receptor signaling pathway | -1.41703 | 0.020938 |
| GO:0061138 | morphogenesis of a branching epithelium | 1.303989 | 0.021089 |
| GO:0048754 | branching morphogenesis of an epithelial tube | 1.352048 | 0.021412 |
| GO:0001659 | temperature homeostasis | -1.37474 | 0.021584 |
| GO:0042593 | glucose homeostasis | -1.35355 | 0.021795 |
| GO:0002688 | regulation of leukocyte chemotaxis | -1.44516 | 0.022113 |
| GO:0046879 | hormone secretion | -1.30775 | 0.022167 |
| GO:0007218 | neuropeptide signaling pathway | -1.44772 | 0.022321 |
| GO:0045785 | positive regulation of cell adhesion | -1.2693 | 0.022673 |
| GO:0072593 | reactive oxygen species metabolic process | -1.31405 | 0.022901 |
| GO:0033500 | carbohydrate homeostasis | -1.35235 | 0.023047 |
| GO:0090276 | regulation of peptide hormone secretion | -1.36053 | 0.023196 |
| GO:0030324 | lung development | 1.309424 | 0.023249 |
| GO:0010810 | regulation of cell-substrate adhesion | -1.36765 | 0.023316 |
| GO:0007498 | mesoderm development | 1.363095 | 0.023511 |
| GO:0072009 | nephron epithelium development | 1.389795 | 0.023968 |
| GO:0045861 | negative regulation of proteolysis | 1.199247 | 0.024999 |
| GO:0006643 | membrane lipid metabolic process | -1.34739 | 0.025773 |
| GO:1901605 | alpha-amino acid metabolic process | 1.28419 | 0.025804 |
| GO:0071326 | cellular response to monosaccharide stimulus | -1.38551 | 0.025899 |
| GO:0042129 | regulation of T cell proliferation | -1.37384 | 0.026099 |
| GO:0045995 | regulation of embryonic development | 1.349699 | 0.026497 |
| GO:0001676 | long-chain fatty acid metabolic process | -1.43811 | 0.026786 |
| GO:0007584 | response to nutrient | -1.32978 | 0.027167 |
| GO:0046631 | alpha-beta T cell activation | -1.38181 | 0.027221 |
| GO:0045727 | positive regulation of translation | 1.383329 | 0.027274 |
| GO:0048638 | regulation of developmental growth | -1.27844 | 0.028015 |
| GO:0009743 | response to carbohydrate | -1.34443 | 0.028133 |
| GO:0030595 | leukocyte chemotaxis | -1.33843 | 0.028133 |
| GO:0071219 | cellular response to molecule of bacterial origin | 1.255628 | 0.028694 |
| GO:0010811 | positive regulation of cell-substrate adhesion | -1.38807 | 0.028777 |
| GO:0010927 | cellular component assembly involved in morphogenesis | -1.39359 | 0.029155 |
| GO:0070085 | glycosylation | -1.29135 | 0.029225 |
| GO:0046467 | membrane lipid biosynthetic process | -1.3659 | 0.029371 |
| GO:0061013 | regulation of mRNA catabolic process | 1.254222 | 0.029478 |
| GO:0030041 | actin filament polymerization | -1.34121 | 0.02969 |
| GO:0031532 | actin cytoskeleton reorganization | -1.40884 | 0.029806 |
| GO:0007229 | integrin-mediated signaling pathway | -1.41141 | 0.029851 |
| GO:0035107 | appendage morphogenesis | 1.324967 | 0.029953 |
| GO:0035108 | limb morphogenesis | 1.324967 | 0.029953 |
| GO:0006022 | aminoglycan metabolic process | -1.33597 | 0.030055 |
| GO:0009749 | response to glucose | -1.33854 | 0.030303 |
| GO:0050680 | negative regulation of epithelial cell proliferation | -1.38402 | 0.030435 |
| GO:0006959 | humoral immune response | 1.242779 | 0.030823 |
| GO:0071222 | cellular response to lipopolysaccharide | 1.281883 | 0.031317 |
| GO:0071331 | cellular response to hexose stimulus | -1.35996 | 0.031609 |
| GO:0016054 | organic acid catabolic process | -1.28861 | 0.031726 |
| GO:0046395 | carboxylic acid catabolic process | -1.28861 | 0.031726 |
| GO:0009914 | hormone transport | -1.2724 | 0.031785 |
| GO:0009755 | hormone-mediated signaling pathway | -1.30724 | 0.032051 |
| GO:0072006 | nephron development | 1.32671 | 0.032059 |
| GO:0007018 | microtubule-based movement | 1.211287 | 0.03231 |
| GO:0031960 | response to corticosteroid | -1.34555 | 0.032877 |
| GO:0055002 | striated muscle cell development | -1.34731 | 0.032877 |
| GO:0006633 | fatty acid biosynthetic process | -1.34738 | 0.033149 |
| GO:0016064 | immunoglobulin mediated immune response | -1.38197 | 0.033237 |
| GO:0042098 | T cell proliferation | -1.3163 | 0.033557 |
| GO:0051604 | protein maturation | 1.201175 | 0.033611 |
| GO:0009746 | response to hexose | -1.33204 | 0.033679 |
| GO:0051053 | negative regulation of DNA metabolic process | 1.313248 | 0.034025 |
| GO:0046883 | regulation of hormone secretion | -1.28367 | 0.034221 |
| GO:0051258 | protein polymerization | -1.28451 | 0.034307 |
| GO:0010508 | positive regulation of autophagy | -1.36981 | 0.034483 |
| GO:0050730 | regulation of peptidyl-tyrosine phosphorylation | -1.28371 | 0.034749 |
| GO:0015893 | drug transport | -1.331 | 0.034794 |
| GO:0000910 | cytokinesis | 1.28337 | 0.034987 |
| GO:0097529 | myeloid leukocyte migration | -1.31688 | 0.035065 |
| GO:0038127 | ERBB signaling pathway | -1.34874 | 0.035261 |
| GO:0002695 | negative regulation of leukocyte activation | -1.33197 | 0.035374 |
| GO:0019724 | B cell mediated immunity | -1.36626 | 0.035868 |
| GO:0045444 | fat cell differentiation | -1.31176 | 0.036082 |
| GO:0048813 | dendrite morphogenesis | -1.33392 | 0.036364 |
| GO:0042326 | negative regulation of phosphorylation | -1.23022 | 0.036471 |
| GO:0022412 | cellular process involved in reproduction in multicellular organism | 1.189719 | 0.036734 |
| GO:0006520 | cellular amino acid metabolic process | 1.156957 | 0.036766 |
| GO:0030203 | glycosaminoglycan metabolic process | -1.33459 | 0.036885 |
| GO:0048640 | negative regulation of developmental growth | -1.3868 | 0.037369 |
| GO:0034284 | response to monosaccharide | -1.31374 | 0.037662 |
| GO:0031589 | cell-substrate adhesion | -1.2453 | 0.038084 |
| GO:0030833 | regulation of actin filament polymerization | -1.32284 | 0.038251 |
| GO:0021782 | glial cell development | -1.38643 | 0.038292 |
| GO:0002460 | adaptive immune response based on somatic recombination of immune receptors built from immunoglobulin superfamily domains | -1.28613 | 0.038363 |
| GO:0007265 | Ras protein signal transduction | -1.2324 | 0.038417 |
| GO:0006650 | glycerophospholipid metabolic process | -1.26537 | 0.039312 |
| GO:0031667 | response to nutrient levels | -1.22095 | 0.039673 |
| GO:0010256 | endomembrane system organization | -1.22161 | 0.039859 |
| GO:0050727 | regulation of inflammatory response | -1.25516 | 0.039952 |
| GO:0060070 | canonical Wnt signaling pathway | 1.189893 | 0.040471 |
| GO:0050907 | detection of chemical stimulus involved in sensory perception | 1.130656 | 0.041014 |
| GO:0010675 | regulation of cellular carbohydrate metabolic process | -1.34547 | 0.041135 |
| GO:0051897 | positive regulation of protein kinase B signaling | -1.31292 | 0.041265 |
| GO:0045765 | regulation of angiogenesis | -1.25751 | 0.041719 |
| GO:0046660 | female sex differentiation | 1.3353 | 0.041726 |
| GO:0006486 | protein glycosylation | -1.27874 | 0.042199 |
| GO:0043413 | macromolecule glycosylation | -1.27874 | 0.042199 |
| GO:0043271 | negative regulation of ion transport | -1.31939 | 0.042641 |
| GO:0060538 | skeletal muscle organ development | -1.32011 | 0.042641 |
| GO:0030308 | negative regulation of cell growth | -1.30458 | 0.042953 |
| GO:0071333 | cellular response to glucose stimulus | -1.33686 | 0.04329 |
| GO:0006414 | translational elongation | 1.312105 | 0.0434 |
| GO:0001823 | mesonephros development | 1.334211 | 0.043538 |
| GO:0030323 | respiratory tube development | 1.276608 | 0.043629 |
| GO:0001666 | response to hypoxia | -1.22853 | 0.04401 |
| GO:0043401 | steroid hormone mediated signaling pathway | -1.3018 | 0.044295 |
| GO:0046488 | phosphatidylinositol metabolic process | -1.3098 | 0.044959 |
| GO:0002449 | lymphocyte mediated immunity | -1.28258 | 0.044987 |
| GO:0050867 | positive regulation of cell activation | -1.24446 | 0.045122 |
| GO:0006304 | DNA modification | 1.316554 | 0.045359 |
| GO:0001678 | cellular glucose homeostasis | -1.31438 | 0.045392 |
| GO:0046777 | protein autophosphorylation | -1.2703 | 0.046095 |
| GO:0051101 | regulation of DNA binding | 1.327407 | 0.046389 |
| GO:1903510 | mucopolysaccharide metabolic process | -1.34887 | 0.047128 |
| GO:0052548 | regulation of endopeptidase activity | 1.161697 | 0.047356 |
| GO:0044272 | sulfur compound biosynthetic process | -1.29668 | 0.048064 |
| GO:0002696 | positive regulation of leukocyte activation | -1.24513 | 0.048148 |
| GO:0035270 | endocrine system development | 1.278385 | 0.048544 |
| GO:0046887 | positive regulation of hormone secretion | 1.297391 | 0.048544 |
| GO:0007601 | visual perception | 1.202515 | 0.048769 |
| GO:0048469 | cell maturation | -1.3008 | 0.048847 |
| GO:0060249 | anatomical structure homeostasis | 1.144939 | 0.048869 |
| GO:0072001 | renal system development | 1.172186 | 0.048915 |
| GO:0007266 | Rho protein signal transduction | -1.29172 | 0.049351 |
| GO:1990138 | neuron projection extension | -1.30423 | 0.049519 |
| GO:0022037 | metencephalon development | -1.34296 | 0.04978 |
